# Bilateral cytomegalovirus (CMV) oophoritis mimicking widely metastatic carcinoma: a case report and review of the literature

**DOI:** 10.1186/1746-1596-2-50

**Published:** 2007-12-20

**Authors:** Jing Yu, Francis X Solano, Raja R Seethala

**Affiliations:** 1 Department of Pathology, University of Pittsburgh Medical Center, Pittsburgh, PA 15213, USA; 2 Solano & Kokales Internal Medicine Associates, University of Pittsburgh Medical Center, Pittsburgh, PA 15213, USA

## Abstract

Ovarian cytomegalovirus (CMV) infection is a rare finding reported in autopsy studies of immunocompromised patients. We report the first case of bilateral CMV oophoritis diagnosed in surgical resection specimens from a 63-year-old woman with metastatic brain lesions undergoing whole brain radiation and steroid treatment. The ovarian involvement of CMV infection was an incidental finding during the colectomy and bilateral salpingo-oophorectomy procedure for gastrointestinal bleeding and presumed ovarian metastases. In contrast to the prevailing dogma, a review of the literature found similar prevalence of pre-menopausal and post-menopausal cases. While age related vasculopathy was thought to be the prevailing mechanism for CMV oophoritis, the observation of an inflammation mediated microthrombosis in our case provides a plausible age independent mechanism suggesting that both restrictive and obstructive vascular changes can be involved in the pathogenesis of CMV oophoritis. To avoid misdiagnosis, both pathologists and clinicians should recognize ovarian involvement by CMV as a possibility in the immunocompromised patient.

## Background

Cytomegalovirus (CMV) oophoritis has been rarely reported in the literature [1-10]. It usually occurs as an incidental finding in immunocompromised patients, either as part of a disseminated infection or as an isolated condition. We report here the first case of bilateral CMV oophoritis diagnosed in surgical resection specimens from a 63-year-old woman with metastatic brain lesions undergoing whole brain radiation and steroid treatment.

## Case presentation

The patient was a 63-year-old woman with a history of lung cancer who presented with a three-week history of confusion and homonymous hemianopsia. On CT scan, she was found to have multiple hypodense cerebral lesions that were presumed to be metastases. She subsequently received whole brain radiation and steroids. Her post treatment course was complicated by heavy gastrointestinal bleeding from a duodenal ulcer. Three weeks subsequent to initiation of therapy, she developed a colonic perforation and underwent emergent laparotomy. The ovaries were also noted to be enlarged bilaterally and thought to be involved by metastatic disease. The patient underwent a left hemicolectomy and bilateral salpingo-oophorectomy. However, the patient's gastrointestinal bleeding persisted, and she passed away four days after surgery. A post-mortem examination was not performed.

### Pathological findings

**Figure 1:**
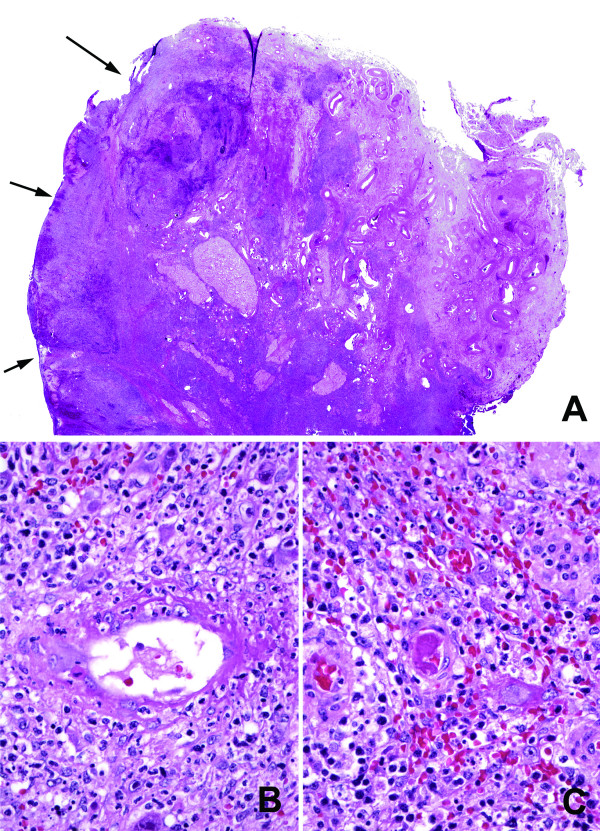
(A) Areas of hemorrhage with extensive coagulative necrosis (arrows) (H&E, whole slide scan, equivalent to magnification of approximately ×12). (B) Vasculitis and (C) Fibrin microthrombus (H&E, original magnifications ×400).

**Figure 2:**
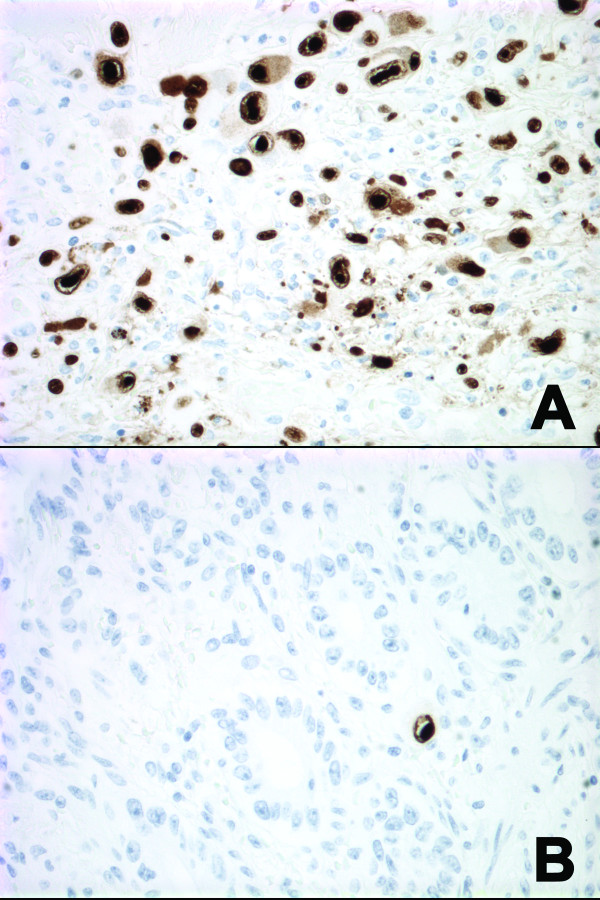
(A) Massive infiltration of ovarian cortex by CMV infected cells and (B) Scattered CMV positive cells in the colon (Immunoperoxidase, original magnification ×400).

On gross examination, both ovaries were edematous and showed cortical hemorrhage. The left ovary also demonstrated a 2.5 cm homogeneous calcific white nodule. Microscopic examination revealed extensive cortical coagulative necrosis within the areas of hemorrhage (Fig. 1A). In these hemorrhagic areas, there was a mixed inflammatory infiltrate accompanied by sheets and clusters of enlarged stromal and endothelial cells with abundant amphophilic cytoplasm, large intranuclear inclusions that formed characteristic perinuclear halos, and variable intracytoplasmic inclusions. Fibrin thrombi were identified within or adjacent to the lesional areas (Fig. 1B–C). An immunohistochemical stain for cytomegalovirus protein (clone DDG9+CCH2, dilution 1:50, Dako Cytomation) highlighted massive infiltration of ovarian cortex by CMV infected cells (Fig. 2A).

**Figure 3:**
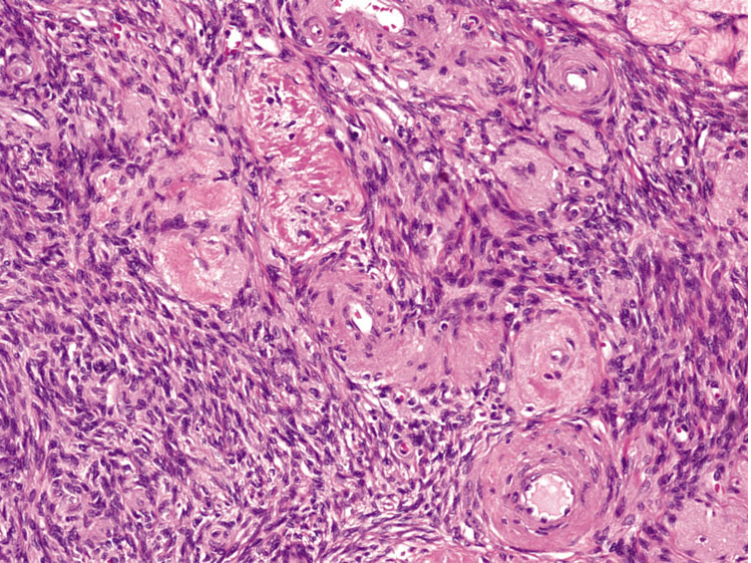
Prominent restrictive vascular changes (H&E, original magnifications ×100).

There were also prominent post-menopausal restrictive vascular changes throughout the surrounding ovary, consisting of markedly thickened arterial walls with deposition of eosinophilic collagenous material (Fig. 3). The left ovary also contained a 2.5 cm ovarian fibroma.

The resected segment of colon demonstrated diverticulitis, including one ruptured diverticulum with a surrounding serositis. The fallopian tubes demonstrated an acute salpingitis. Immunohistochemical stains for CMV highlighted scattered positive cells in the lamina propria as well as in the serosa of the colon near the perforation (Fig. 2B).

## Discussion

Previously, there have been 10 reports of 12 cases of CMV oophoritis in the English literature [1-10]. The main clinical features of all cases including the current case are summarized in Table 1. The mean age at presentation was 44.7 (range: 11–67), and 7/13 (53.8%) were post-menopausal. All patients showed evidence of immunosuppression. Eight of thirteen (61.5%) had pre- or post-mortem documentation of systemic involvement by CMV and two of these cases (2/8, 25%) received ganciclovir as treatment. Five of thirteen (38.5%) had a clinical or gross presentation of mass lesion (s).

Originally described by Subietas et al in 1977 [8], bilateral CMV oophoritis is a rare pathologic finding that is usually associated with a systemic infection. An underlying malignancy or immunosuppressive status is almost always present and steroid therapy has been shown to be a strong risk factor for CMV reactivation in both groups [5,8,11], as it is in our current case. It has been suggested that previously reported isolated CMV oophoritis might represent sampling error [7]. Indeed, the presence of a duodenal ulcer also raises the possibility that this incidental finding of bilateral oophoritis might have been part of a generalized CMV infection. However, there was no serologic or post-mortem documentation of systemic disease in this case.

It is worth noting that only slightly over half of the reported CMV oophoritis cases were from post-menopausal women [1-3,5,9,10], even though it was historically thought to be a disease of post-menopausal patients [4,6-8]. This revised age distribution has some implications with regards to pathogenesis. The predilection of CMV for endothelial cells is thought to be important in the pathogenesis of CMV oophoritis. Initially, an explanation of the predilection of CMV oophoritis towards the post menopausal age was that the obstruction of blood flow secondary to age related restrictive vascular changes seen in post-menopausal women was a critical factor for localizing the infection to the ovary, thus allowing progression of disease [3,8,10]. The ovaries in our current case did indeed demonstrate prominent age related vascular changes in the post-menopausal ovaries, including eosinophilic collagen deposition and vessel wall thickening leading to narrowed lumina.

However, the subsequent inflammatory response and secondary vasculitis with microthrombi seen in our case also likely perpetuated this vascular restriction. CMV infected endothelial cells have been observed in the small vessels of the cortex in almost every reported case of CMV oophoritis, in both pre- and post-menopausal women (1–10). Generalized endothelial cell involvement in small vessels is a common phenomenon in multi-organ CMV disease [3]. Iwasaki et al. suggested that tissue involvement might begin with endothelial cell infection after the virus enters the ovary through bloodstream. The infected endothelial cells lining the vascular wall or those "desquamated" into the vascular spaces might have caused vascular obstruction, resulting in a localized vasculitis, thrombosis and secondary distal coagulative necrosis.

Additionally, mounting evidence suggests that CMV virus particles can directly initiate coagulation cascade and thrombin production [12,13]. In vitro studies suggest the activation of coagulation system mediated by tissue factor expression on the surface of infected endothelial cells, in combination with the essential phospholipids and tissue factor activity on modified CMV envelope [12,13]. In vivo studies demonstrate CMV-induced high factor VIII concentration and increased von Willebrand factor levels, suggesting a procoagulant response [14,15]. Numerous cases of acute thrombosis during CMV infection have been reported, manifested as either disseminated intravascular coagulation or localized organ specific infarct [12,16].

The observation of multiple small vessel fibrin thromboemboli as seen in our case was not mentioned as a contributing factor in any of the previously reported CMV oophoritis cases. In fact, the lack of microthrombi was noted by Subietas et al [8] and actually considered one of the arguments against a virally induced vasculopathy. However, our case provides evidence for an alternate pathway for perpetuation of CMV infection in the ovary that does not require post menopausal restrictive vascular changes, thus providing one possible explanation for the nearly equal distribution between pre- and post- menopausal patients [1-3,5,9,10].

The vast majority of reported cases were missed during the patients' lifetimes, except one case that was diagnosed in a patient who underwent surgical procedure for CMV-associated pelvic infectious disease and the ovarian involvement was limited to the left ovary [9]. Our current case is the first reported case of bilateral CMV oophoritis diagnosed in a surgical specimen for a patient with non-gynecological disorders. Subietas et al [8] and LiVolsi and Merino [4] have previously emphasized the diagnostic challenge of CMV oophoritis both clinically and pathologically. Difficulties in diagnosis arise from inadequate sampling of macroscopically normal-sized ovaries, the assumption that enlarged ovaries are involved by metastatic disease, particularly when there is a known concurrent malignancy, and the similar microscopic appearance of massive CMV infection to a malignant infiltrate. The ovarian changes in our case were initially presumed to be metastatic tumors by the surgeon. In fact, 38.5% of previously reported cases did indeed grossly mimic tumors or mass lesions. Despite the lack of direct symptomatology from the oophoritis, all reported cases demonstrated cortical destruction and extensive histologic involvement by CMV infected cells. The cells enlarged by both nuclear and cytoplasmic inclusions may mimic an anaplastic carcinoma, melanoma or a large cell lymphoma, and thus awareness of the entity of CMV oophoritis is tantamount to its recognition [3,8].

It is unclear whether ovarian CMV infection is a manifestation of systemic infection or actually the primary site of involvement. The putative vascular/restrictive pathogenesis model described above along with cases of isolated CMV oophoritis [5,7] make the ovaries plausible candidates to serve as CMV reservoirs. Additionally, CMV DNA has been identified in arterial wall smooth muscle cells in both seropositive and seronegative healthy individuals [17]. However, the fact remains that most CMV oophoritis cases have been associated with concurrent multiorgan involvement, making it difficult to make any conclusive statements regarding CMV manifestation in the ovary [1-3,6,8,10]. Furthermore, data on the role of the ovary in CMV latency is sparse [17-20].

Antiviral agents such as ganciclovir are the treatment of choice for CMV infection. In the two cases that had pre-mortem documentation of systemic CMV infection and were treated with ganciclovir, post-mortem studies revealed continuing systemic infection in one case [2] and an isolated CMV oophoritis in the other case [6] (Table 1). The residual isolated CMV oophoritis after ganciclovir treatment illustrated the resistance to ganciclovir in the ovaries. It has been postulated that decreased blood flow in the postmenopausal ovary might reduce the ganciclovir level in ovarian tissue and help sequester CMV from antiviral treatment [6].

**Table 1:** Main clinical features of reported CMV oophoritis cases in the English literature

First Author	Age	Menopausal Status	Primary Diagnosis	Prior Immunosuppressive Treatment/State	Documented Systemic CMV infection	Treatment of CMV	Presentatation as Mass lesion	Diagnostic Material
Subietas (1977)	62	Post-menopausal	Astrocytoma	Radiation	Yes (post-mortem)	No	No	Autopsy
Subietas (1977)	40	Post-menopausal	Hodgkin Lymphoma,	Chemotherapy, Steroids, Radiation	Yes (post-mortem)	No	No	Autopsy
Subietas (1977)	67	Post-menopausal	Breast CA	Testosterone, Steroids	No	No	No	Autopsy
Evans (1978)	37	Pre-menopausal	SLE	Steroids	Yes (post-mortem)	No	Yes	Autopsy
LiVolsi (1979)	61	Post-menopausal	Lymphoma	Chemotherapy, Steroids	Yes (post-mortem)	No	No	Autopsy
Iwasaki (1988)	11	Pre-pubertal	ALL	Chemotherapy, Steroids	Yes (pre-mortem)	N/A	No	Autopsy
Williams (1989)	40	Pre-menopausal	Cholangio-CA	Liver Transplant	Yes (post-mortem)	No	Yes	Autopsy
Familiari (1990)	33	Pre-menopausal	AIDS	Anti-retrovirals	Yes (pre-mortem)	Yes	No	Autopsy
Sharma (1994)	50	Post-menopausal	Breast CA	Autologous BMT	No	No	No	Autopsy
Wales (1996)	31	Pre-menopausal	HIV, PID	Anti-retrovirals,	No	No	Yes (limited to left ovary)	Surgical Resection
Nieto (1999)	50	Post-menopausal	Breast CA	Allogenic BMT	Yes (pre-mortem) *	Yes	No	Autopsy
Manfredi (2000)	36	Pre-menopausal	AIDS	Presumed CNS Toxoplasmosis & Lymphoma	No	No	Yes	Autopsy
Current Case (2007)	63	Post-menopausal	Lung CA	Radiation, Steroids	No	No	Yes	Surgical Resection

ALL: acute lymphocytic leukemia; BMT: bone marrow transplantation; CA: carcinoma; CNS: central nervous system; PID: pelvic infectious disease; SLE: systemic lupus erythematosus; N/A: not available; *: pre-mortem systemic CMV infection but post-mortem isolated oophoritis

Hence in summary, CMV oophoritis is a rare occurrence that may affect both post- and pre-menopausal women who are immunocompromised. Observations of this case suggest that vascular changes, both native (restrictive) and virally induced (obstructive), represent viable pathogenetic mechanisms. Despite the lack of direct symptomatology, CMV oophoritis often presents as a mass lesion, and characteristically shows extensive cortical destruction and a high burden of virally infected cells. However, it is still unclear whether CMV reactivation in the ovaries is only a component of systemic spread or the actual primary site of origin of disease. To avoid misdiagnosis or underdiagnosis, both pathologists and clinicians should recognize ovarian involvement by CMV as a possibility in the immunocompromised patient.

## Competing interests

The author(s) declare that they have no competing interests.

## Authors' contributions

All authors have contributed to the content and design of this study.
